# Autologous serum eye drops diluted with 0.5% methylcellulose and 0.9% saline solution: a clinical comparative study

**DOI:** 10.5935/0004-2749.2022-0064

**Published:** 2023-03-20

**Authors:** Nessana Neubauer, Victória d‘Azeredo Silveira, Claudete Ines Locatelli, Diane Ruschel Marinho

**Affiliations:** 1 Postgraduate Program in Medicine: Surgical Sciences, Faculdade de Medicina, Universidade Federal do Rio Grande do Sul, Porto Alegre, RS, Brazil; 2 Department of Ophthalmology, Hospital de Clínicas de Porto Alegre, Porto Alegre, RS, Brazil; 3 Department of Ophthalmology, Faculdade de Medicina, Universidade Federal do Rio Grande do Sul, Porto Alegre, RS, Brazil.

**Keywords:** Dry eye syndromes, Keratoconjunctivitis sicca, Sjögren’s syndrome, Saline solution, Ophthalmic solutions, Methylcellulose, Síndromes do olho seco, Ceratoconjuntivite seca, Síndrome de Sjögren, Solução salina, Soluções oftálmicas, Metilcelulose

## Abstract

**Purpose:**

This clinical study compared autologous serum eye drops diluted with 0.5% methylcellulose and 0.9% saline solution. The subjective criteria for symptom improvement and the objective clinical criteria for response to therapy were evaluated.

**Methods:**

This longitudinal prospective study enrolled 23 patients (42 eyes) with persistent epithelial defects or severe dry eye disease refractory to conventional therapy who had been using autologous serum 20% prepared with methylcellulose for > 6 months and started on autologous serum diluted in 0.9% saline solution. The control and intervention groups consisted of the same patients under alternate treatments. The subjective criteria for symptom relief were evaluated using the Salisbury Eye Evaluation Questionnaire. The objective clinical criteria were evaluated through a slit-lamp examination of the ocular surface, tear breakup time, corneal fluorescein staining, Schirmer’s test, rose Bengal test, and tear meniscus height. These criteria were evaluated before the diluent was changed and after 30, 90, and 180 days.

**Results:**

In total, 42 eyes were analyzed before and after 6 months using autologous serum diluted with 0.9% saline. No significant differences were found in the subjective criteria, tear breakup time, tear meniscus, corneal fluorescein staining, or rose Bengal test. Schirmer’s test scores significantly worsened at 30 and 90 days (p=0.008). No complications or adverse effects were observed.

**Conclusions:**

This study reinforces the use of autologous serum 20% as a successful treatment for severe dry eye disease resistant to conventional therapy. Autologous serum in 0.9% saline was not inferior to the methylcellulose formulation and is much more cost-effective.

## Abstract (pt)

**Objetivo:**

Este estudo comparou o colírio de soro au tólogo manipulado com metilcelulose a 0,5% com solução salina 0,9%. Critérios subjetivos de melhora dos sintomas e critérios clínicos objetivos para resposta à terapia foram avaliados.

**Métodos:**

Este estudo prospectivo longitudinal envolveu 23 pacientes (42 olhos) com defeitos epiteliais persistentes ou doença de olho seco grave refratária à terapia convencional que usavam colírio de soro autólogo 20% preparado com metilcelulose por mais de 6 meses e iniciaram soro autólogo diluído em solução salina 0,9%. Os grupos controle e intervenção consistiam dos mesmos pacientes sob tratamentos alternados. Os critérios subjetivos para o alívio dos sintomas foram avaliados usando o *Salisbury Eye Evaluation Questionnaire*. Os critérios objetivos foram avaliados por meio de exame em lâmpada de fenda incluindo: tempo de ruptura da lágrima, coloração da córnea com fluoresceína, teste de Schirmer, coloração com rosa bengala e altura do menisco lacrimal. Esses critérios foram avaliados antes da troca do diluente e após 30, 90 e 180 dias.

**Resultados:**

Um total de 42 olhos foram analisados antes e após 6 meses usando soro autólogo diluído com solução salina 0,9%. Nenhuma diferença significativa foi encontrada nos critérios subjetivos, tempo de ruptura da lágrima, menisco lacrimal, coloração com fluoresceína ou rosa bengala. Os resultados dos testes de Schirmer pioraram significativamente em 30 e 90 dias (p=0,008). Não foram observadas complicações ou efeitos adversos.

**Conclusões:**

Este estudo reforça o uso do colírio de soro autólogo 20% como um tratamento de sucesso para a doença do olho seco grave resistente à terapia convencional. O soro autólogo diluído em solução salina a 0,9% não foi inferior à formulação de metilcelulose.

## INTRODUCTION

Dry eye syndrome is a multifactorial disorder that affects tear function and the ocular surface. Epidemiological studies have concluded that the prevalence of dry eye can range from 5% to 30% in individuals aged > 50 years. This condition was estimated to affect approximately 12.9% of Brazilians. Severe dry eye is commonly associated with conditions such as persistent epithelial defect, neurotrophic ulcers, ocular cicatricial pemphigoid, Stevens-Johnson syndrome, Sjögren syndrome, post-corneal transplant, post-LASIK, herpetic keratitis, graft-versus-host disease, and post-irradiation keratopathy^(1,2,3)^.

Biological fluids have been increasingly investigated and advocated in the treatment of ocular pathologies. Autologous serum (AS) has been used to treat patients with dry eye since 1984 when Fox et al.^(4)^ attempted to produce preservative-free artificial tears that would both lubricate the eye and provide other essential components of natural tears^(5,6)^.

Eye drops produced from AS have been reported as an effective alternative for moderate-to-severe cases of dry eye resistant to conventional medical therapy, such as artificial tears, topical cyclosporine, and/or lacrimal duct occlusion. The biochemical and biomechanical properties of preparations made from human AS similar to those of natural tears, providing real epithelia tropic potential for the proliferation, migration, and differentiation of epithelial cells on the ocular surface, which could contribute to better wound healing after surgical interventions or in persistent corneal ulcers^(3,4,5,6)^.

In literature, the traditional and first described formulations of AS 20% were diluted with saline solution (SS). Some authors have described the use of other diluents such as balanced SS (BSS) and sodium hyaluronate to obtain even better results. The only comparative study between SS and hyaluronate found comparable clinical results and concluded that preparations diluted with sodium hyaluronate were better tolerated by patients and require a few drops^(7)^.

In our hospital, we used to dilute AS in 0.5% methylcellulose. However, the cost of each 10-mL bottle of methylcellulose is BRL 39.80 (approximately USD 7.39), and since AS can be prepared in 0.9% SS, which costs approximately BRL 5.00 (USD 0.93) per liter, this alternative appeared much more cost-effective^(8,9,10,11,12)^.

We hypothesized that formulating AS in 0.9% SS rather than methylcellulose would not affect the results, which would considerably reduce production costs and, thus, patient costs. The issue of cost-effectiveness is extremely important because in many cases, AS drops may be required permanently. Therefore, this study aimed to compare the results of using AS in 0.5% methylcellulose with 0.9% SS.

## METHODS

### Study design

This prospective longitudinal study assessed patients who had been using AS 20% prepared with methylcellulose for > 6 months and started on AS diluted in 0.9% SS. The control and intervention groups consisted of the same patients in alternate treatments.

### Participants

The study participants were patients with severe dry eye resistant to conventional therapy, including artificial tears, anti-inflammatory drops, lacrimal duct occlusion, and/or therapeutic contact lenses who were using AS diluted in methylcellulose and had a stable ocular surface. Most of the patients had associated comorbidities. The exclusion criteria were as follows: treatment inadherence, systemic infection or infection at the arm puncture site, and epithelial defect with imminent perforation that required surgical treatment.

Data on AS in 0.9% SS are described here according to the objective and subjective description after 6 months of monitoring. The patients were analyzed on the first day of treatment and again after 30, 90, and 180 days. Besides these consultations, they came in monthly to receive a batch of 30 bottles of AS.

### Evaluation and continuity

a) Subjective criteria: The Salisbury Eye Evaluation Questionnaire on dry eye symptoms was answered before the intervention and again after 30, 90, and 180 days of treatment with AS diluted with SS. Patients with two or more positive answers were considered symptomatic.
b) Objective criteria: A full ophthalmological examination and evaluation of the ocular surface with biomicroscopy, tear breakup time, corneal fluorescein staining, Schirmer’s test, rose Bengal test, and tear meniscus evaluation. Biomicroscopy evaluated eyelid alterations, presence of symblepharon, and transparency and vascularization of the corneal surface. The tear breakup time was tested conventionally, using a wet stick with fluorescein on the bottom of the conjunctival fornix. The average of three measurements was taken, and a time over 10 s was considered normal. Corneal staining by fluorescein was scored from 0 to 3: 0, no stain; 1, less than ⅓ of the cornea stained; 2, ⅓ to ⅔ of the cornea stained; 3, more than ⅔ of the cornea stained. Scores of 0 and 1 were considered normal. To evaluate basal tear production, Schirmer’s test was performed with anesthetic, and the eyes closed. A standard filter paper (5 mm × 35 mm, with a fold 5 mm from the edge) was placed in the lateral third of the lower edge of the eyelid. After 5 min, the quantity of moisture absorbed by the paper was verified, with > 5 mm considered normal. Conjunctival corneal staining was graded from 0 to 9 after instillation of 1% rose Bengal eye drops, according to the Van Bijsterveld scoring system. Staining was graded by summing the scores (0-3) from the medial bulbar conjunctiva, cornea, and temporal bulbar conjunctiva^(13)^. A tear meniscus ≥3 mm was considered normal.

### Data analysis

Data were analyzed using generalized estimating equations, considering binary data and logit links. For statistical analysis, the responses were dichotomized, with 0 considered normal and 1 abnormal. A robust estimation concordance matrix and an unstructured working correlation matrix were used. For comparison, the categories were divided into the presence or absence of mild symptoms and the presence or absence of moderate/severe symptoms. The significance level was set at 0.05, and post-hoc Bonferroni tests were used when significant differences were found. Since the analysis involved repeated measures (i.e., the same patient assessed at four points), we used statistical tests for related samples. Thus, generalized estimating equations were used to compare the probability of dry eye symptoms and objective criteria at these points. Data were presented as probability and 95% confidence interval. The analyses were performed in SPSS Statistics version 18 (SPSS Inc., Chicago, IL, USA).

### AS preparation

In conformity with Hospital de Clínicas de Porto Alegre Infection Control Committee, the AS was prepared in the hospital’s Molecular Analysis of Proteins Unit, and blood collection was performed at the hospital’s Clinical Research Center as follows:

- A total of 120 mL of blood was collected from each patient in thirty 4 mL siliconized flasks. The blood was immediately centrifuged at 2,500 rpm for 10 min at room temperature.
- Then, using laminar-flow cabinets, 1 mL of the serum was withdrawn from the flask and added to 4 mL of preservative-free 0.9% SS, thus obtaining 60 bottles of sterile 20% AS eye drops, each containing approximately 2-3 mL.
- A sample (2 mL) was stored in a sterile bottle and sent to the microbiology lab for the microbiological analysis.
- Each culture sample was identified with the patient’s name, preparation date, batch number, and name of the technician who produced it.
- The bottles of AS eye drops were stored in a Styrofoam box that had been previously cleaned with 70% alcohol.
- The AS bottles were kept in a freezer at -20°C at the Molecular Analysis of Proteins Unit until they were delivered to the patient (stored in Styrofoam box with ice) after the microbiology lab results were released.
- All microbiological analysis results were kept on file.
- The patients were instructed to keep the bottles properly closed and stored in a freezer at -20°C. Only the bottle in use could be kept in the refrigerator at 2°C-8°C. They were also instructed to use a bottle for a maximum of 48 h, unless the drops changed color or smell, in which case, the bottle was to be discarded.
- The participants used the eye drops every hour or every 2 hours daily.

### Ethical issues

All patients provided written informed consent before enrollment, being aware of the objectives and risks of the study and allowing the use of their clinical data. The patients’ identities remain anonymous. The study was approved by the *Hospital de Clínicas de Porto Alegre* Research Ethics Committee (CAEE 54743315.3.00000.5327).

## RESULTS

A total of 26 patients were initially included in the study. During the follow-up period, three losses were incurred, two due to interruption of treatment because of poor adherence and the other due to hospital admission. Thus, 23 patients completed the study, of which 14 were women (60.8%), with age ranging from 33 to 82 (57.5 ± 24.5) and 21 to 72 (46.5 ± 25.5) for women and men, respectively. Nine patients had Sjögren syndrome (42% of the eyes), followed by 5 (21%) patients with Stevens- Johnson syndrome, and 2 (7%) with chemical burns; 30% of the eyes had other associated conditions. The demographic characteristics of the patients are summarized in table 1.

**Table 1:** Demographic characteristics of the sample.

Eye(s) evaluated	Age	Sex	Diagnosis	Comorbidities
OR OL	58	Female	Sjögren syndrome	Rheumatoid arthritis
	72	Female	Sjögren syndrome	Hypothyroidism, Rheumatoid arthritis, dyslipidemia, psoriasis, ocular cicatricial pemphigoid
OR, OL	58	Female	Acanthamoeba keratitis	Hypertension, breast cancer, endometrial cancer, thyroidectomy, corneal transplant
OL	34	Male	Ectrodactyly ectodermal dysplasia (EEC syndrome)	–
OR, OL	82	Female	Sjögren syndrome	Bronchitis and hypertension
OR, OL	37	Male	Stevens-Johnson syndrome	
OR, OL	65	Female	Sjögren syndrome	Hypertension and hypothyroidism
OR, OL	42	Female	Sjögren syndrome	---
OR, OL	48	Female	Sjögren syndrome	Rheumatoid arthritis and hypothyroidism
OR, OL	81	Female	Herpetic ulcer	Glaucoma and rheumatoid arthritis
OR, OL	46	Female	Sjögren syndrome	Depression and dyslipidemia
OR, OL	62	Female	Stevens-Johnson syndrome	---
OR, OL	48	Male	Stevens-Johnson syndrome	---
OR, OL	52	Male	Sjögren syndrome	Hypertension, labyrinthitis, depression, and oral cancer
OR, OL	65	Female	Progressive systemic sclerosis	Hypertension and glaucoma
OR, OL	33	Female	Congenital glaucoma	Cataract, myopia, and toxoplasmosis uveitis
OR	65	Male	Stevens–Johnson syndrome	Stroke
OR, OL	46	Male	Chemical burns	Herpes simplex and herpes zoster infection
OR, OL	28	Male	Aniridia	Mental retardation and depression
OR, OL	36	Female	Budd–Chiari syndrome	Chronic thyroiditis
OR, OL	72	Male	Sjögren syndrome	Cataracts
OL	21	Male	Chemical burns	–
OL	60	Female	Stevens–Johnson syndrome	Hypertension, glaucoma, and corneal transplant

The data of 42 eyes was analyzed over 6 months of follow-up after we began producing AS in 0.9% SS. No significant differences were found in subjective criteria, tear breakup time, tear meniscus, staining with fluorescein, or rose Bengal test results (p > 0.05). However, Schirmer’s test results worsened significantly during the evaluation at days 30 and 90 (p=0.008). The results are presented in tables 2 and 3.

**Table 2:** Six-month assessment results after switching diluent from methylcellulose to 0.9% saline solution

Clinical parameters	Inclusion n (%)	30 days n (%)	90 days n (%)	180 days n (%)	p-value
Subjective questionnaire					
<2 +	10 (24.4)	14 (34.1)	17 (41.5)	8 (19.6)	0.219
≥2 +	31 (75.6)	27 (65.9)	24 (58.5)	33 (80.5)	
Schirmer’s test					
Normal (≥5 mm)	22 (53.7)	18 (43.9)	12 (29.3)	21 (51.2)	0.008
Abnormal (<5 mm)	19 (46.3)	23 (56.1)	29 (70.7)	20 (48.8)	
Fluorescein					
Staining<1/3	17 (41.5)	23 (56.1)	24 (58.5)	24 (58.5)	0.072
Staining ≥1/3	24 (58.5)	18 (43.9)	17(41.5)	17 (41.5)	
Tear breakup time					
Normal (≥10 s)	3 (7.3)	5 (12.2)	4 (9.8)	3 (7.3)	0.832
Abnormal (<10 s)	38 (92.7)	36 (87.8)	37 (90.2)	38 (92.7)	
Tear meniscus					
Normal (≥3 mm)	21 (51.2)	21 (51.2)	16 (39)	23 (56.1)	0.230
Abnormal (<3 mm)	20 (48.8)	20 (48.8)	25 (61)	18 (43.9)	
Rose Bengal					
Mild (<1/3)	19 (46.3)	14 (34.1)	15 (36.6)	16 (39)	0.454
Moderate/severe (1/3)	22 (53.7)	27 (65.9)	26 (63.4)	25 (61)	

**Table 3:** Average estimates of the objective clinical parameters of dry eyes categorized by time

Clinical parameters	Inclusion	Mean %	Standard error	95% Wald Confidence interval	
				Lower	Upper
Abnormal Schirmer’s test (< 5 mm)	Inclusion	46.3	0.078	0.31	0.61
	30 days	56.1	0.078	0.40	0.71
	90 days	70.7	0.071	0. 56	0.84
	180 days	48.8	0.078	0.35	0.64
Fluorescein (≥1/3)	Inclusion	59	0.077	0.43	0.74
	30 days	44	0.078	0.29	0.59
	90 days	41	0.077	0.26	0.57
	180 days	41	0.077	0.26	0.57
Tear breakup time (< 10 seconds)	Inclusion	93	0.041	0.85	1.01
	30 days	88	0.051	0.78	0.98
	90 days	90	0.046	0.81	0.99
	180 days	93	0.041	0.85	1.01
Tear meniscus (< 3 mm)	Inclusion	49	0.078	0.33	0.64
	30 days	49	0.078	0.33	0.64
	90 days	61	0.076	0.46	0.76
	180 days	44	0.078	0.29	0.59
Rose Bengal	Inclusion	54	0.078	0.38	0.69
Moderate/severe	30 days	66	0.074	0.51	0.80
(≥ 1/3 eye)	90 days	63	0.075	0.49	0.78
	180 days	61	0.076	0.46	0.76

## DISCUSSION

Both a deficiency of and structural alterations to the tears lead to important changes, resulting in severe complications for the ocular surface^(6,9)^. In ophthalmology, AS was introduced as an alternative to artificial tears by Fox et al.^(4)^ in 1984. Its use stems from the need for a substitute that, besides lubricating, could provide other essential components of tears. AS also contains immunoglobulins and lysozymes, which give it a bactericidal and bacteriostatic effect, pH, and osmolarity characteristics very similar to natural tears. According to the literature, these features can be obtained by 20% AS in BSS or 0.9% SS. The standard dilution of 20% is based on the concentration of certain growth factors contained in tears, and it is believed that higher concentrations would cause ocular irritation^(3,5,6,8,9,10,11,12,14,15)^.

In 2014, Lopez-Garcia et al. compared AS diluted with sodium hyaluronate and SS and found significantly better results with AS diluted with sodium hyaluronate^(7)^. On the contrary, hyaluronate is two times more expensive than methylcellulose what can influence even more in the final costs of the AS eye drops. In the present study, AS in 0.9% SS was not inferior to methylcellulose; however, patients required more instillations during the day.

Objective evaluation with staining tests (fluorescein and rose Bengal), Schirmer’s test, and impression cytology show improved scores after using AS. A randomized clinical trial by Kojima et al. indicated improvement in the objective and subjective criteria for dry eye after AS treatment compared with preservative-free artificial tears^(3,4,5,7,13,14,15,16)^.

Although no significant differences were found between AS and other conventional therapies (artificial tears or BSS) in some studies, studies of epithelial cell cultures have shown that AS preserved the integrity of cellular membranes and resulted in better intracellular adenosine triphosphate levels than artificial tears. In addition, interrupting AS treatment caused these effects to disappear from the epithelial surface. Although the persistent beneficial effects of AS on epithelial cells can be observed after 2 weeks, patients commonly report subjective improvements by day 2 of treatment^(8,14,17,18)^.

Regarding potential adverse effects, a clinical and in vitro study assessing the use of 20% AS for dry eye and epithelial defects showed low toxicity compared with artificial tears, mainly due to the lack of preservatives. Because bacterial contamination during production is a potential risk, a microbiological examination must be performed before administering the AS eye drops. Although uncommon, some patients can present discomfort or eyelid eczema^(3,9,19)^.

After following 42 eyes with severe dry eye that had been treated with 20% AS in 0.5% methylcellulose for 6 months, 59% of the patients reported symptom improvement, and the majority of clinical dry eye scores improved (p > 0.001). Our service first began producing 20% AS in 0.5% methylcellulose because it is a widely used lubricant in ocular surface diseases and adding a more viscous diluent would improve the results of conventional AS^(11,12)^.

Since we work in a public hospital that treats very poor patients and because 0.9% SS is approximately 160 times cheaper than methylcellulose, we changed the AS dilution to 0.9% SS. After 6 months of follow-up, no significant differences in subjective parameters or majority of the clinical parameters were found between the two formulations. Only the results of Schirmer’s test worsened with 0.9% SS in relation to methylcellulose, which was observed on days 30 and 90. However, if the results were analyzed only at inclusion and on day 180 of treatment with AS in 0.9% SS, there would have been no difference between the formulations. Thus, we believe that with longer follow-up, no significant differences would have been found in any assessed parameter.

Considering the absence of significant changes in the results, the SS alternative is much more cost-effective. The majority of the patients reported no differences after the formula was changed, whereas some reported improvements in symptoms and comfort with 0.9% SS. In addition, all enrolled patients had already tried several other dry eye treatments with no success and reported being dependent on AS to perform their daily living activities.

Depending on the severity and responsiveness to common treatments, AS may be required permanently. Thus, reducing costs allows better access to AS because this treatment is not offered by the Brazilian public health system or covered by health insurance, which means that all expenses are billed to the patient.

AS eye drops appear to be an efficient therapeutic option for treating patients with severe dry eye, mainly in cases unresponsive to other treatments. We found that AS drops diluted in SS were just as good as those diluted with methylcellulose; changing to SS did not decrease the benefits of AS in the same group of patients.
